# Dental fluorosis in the European roe deer (*Capreolus capreolus*): A review of the pathological changes in the enamel of fluorotic cheek teeth and the abnormal pattern of dental wear in affected dentitions

**DOI:** 10.1002/ar.25664

**Published:** 2025-04-01

**Authors:** Uwe Kierdorf, Horst Kierdorf

**Affiliations:** ^1^ Department of Biology University of Hildesheim Hildesheim Germany

**Keywords:** bioindication, dental pathology, enamel mineralization and microstructure, fluoride toxicosis, wildlife toxicology

## Abstract

This article reviews the pathological changes in the enamel of permanent mandibular cheek teeth and their sequelae in European roe deer from regions polluted by anthropogenic fluoride emissions. The primary (developmental) changes of fluorotic roe deer enamel are hypomineralization and microstructural aberrations, including enamel hypoplasia. The hypomineralized fluorotic enamel is whitish‐opaque on eruption and gets stained following tooth eruption. Moreover, it is prone to excessive wear and mechanical breakdown, resulting in posteruptive enamel lesions. These posteruptive lesions can morphologically clearly be distinguished from hypoplastic defects. Due to the impaired mineralization of fluorotic enamel, affected cheek teeth lack the prominent enamel ridges normally present on the occlusal surface. The severity of dental fluorosis typically varies among the permanent mandibular cheek teeth of more severely fluorotic dentitions. While the permanent premolars and the third molar show marked pathological changes, the first molar is largely unaffected, and the second molar is less affected by fluorotic alterations. These differences have been related to protective mechanisms (placental barrier and milk‐blood barrier to fluoride) operating during prenatal and early postnatal life that prevent excessive plasma fluoride levels during crown formation of the M_1_ and partly also of the M_2_ in individuals from fluoride‐polluted habitats. Observations on the fluoride content of early‐formed and late‐formed dentin of cheek teeth from individuals with severe dental fluorosis support this hypothesis. The findings in the European roe deer constitute the most comprehensive characterization of dental fluorosis currently available for a wild ruminant species.

## 1 INTRODUCTION

The European roe deer (*Capreolus capreolus*) is Europe's most abundant wild ruminant species (Lorenzini et al., 2022) and is widely used as a bioindicator of environmental pollution (Kalisińska, 2019; Tataruch & Kierdorf, 2003). Several studies have addressed the effects of chronic overexposure to fluoride from anthropogenic sources on the developing dentition in this species and the use of the resulting dental changes to monitor fluoride pollution of roe deer habitats (Jelenko et al., 2014; Kierdorf, 1988; Kierdorf et al., 1993, 1997, 1999, 2012; Kierdorf & Kierdorf, 1989b, 1997, 2000; Vikøren & Stuve, 1996; Zemek et al., 2007).

Following the characterization of (a) cheek tooth function in ruminants, (b) the permanent dentition of the European roe deer, (c) fluorine in the environment, and (d) the mechanism of dental fluorosis, the present paper reviews the structural changes in fluorotic dental enamel and their sequelae and the variability of fluorosis severity in the permanent cheek teeth of this species. All roe deer dentitions studied originated from free‐ranging individuals and had been collected by hunters during regular management operations in different regions of Central Europe (Jelenko et al., 2014; Kierdorf, 1988; Kierdorf et al., 1999, 2012; Zemek et al., 2007).

## 2 CHEEK TOOTH FUNCTION IN RUMINANTS

Deer and other ruminants rely on mastication as a means of processing their food for subsequent microbial breakdown and passage through the digestive tract. The reduction of particle size of the ingested plant material increases the surface area for the ruminal fermentation process and reduces the retention time of the digesta (Codron et al., 2019).

The functional occlusal relief of ruminant cheek teeth, which are responsible for mastication, consists of elevated enamel cutting edges (ridges) and dentinal troughs or valleys and develops by the wearing away of the enamel initially present at the cusp tips. Ruminant cheek teeth are thus characterized by a so‐called secondary functional crown shape (Fortelius, 1985; Lucas, 2004; O'Brien et al., 2014). The formation of enamel ridges protruding above the dentin on the occlusal surfaces is due to the higher degree of mineralization and the resulting higher wear resistance of enamel compared to dentin (Hillson, 2005; Kierdorf & Becher, 1997; Pérez‐Barbería, 2019). As long as this occlusal relief of the cheek teeth is maintained, the animals are able to effectively process their food.

The mandible of ruminants is narrower than the maxilla, and the left and right sides of the dentition therefore cannot come into occlusion simultaneously. This condition is known as anisognathy and results in unilateral mastication, which is performed by transverse movements of the mandible (Fortelius, 1985; Hiiemae, 1978; Ungar, 2010). During the power stroke of mastication, the mandibular cheek teeth slide over their maxillary antagonists from lateral to medial, and the plant material is subjected to slicing and grinding between the enamel ridges on the opposing occlusal surfaces (Bungo et al., 1999; Fortelius, 1985; Hiiemae, 1978).

Except for species with ever‐growing teeth (hypselodont condition), a progressive reduction in dental crown height from attrition and abrasion and a gradual loss of dental functionality due to wear occur in all herbivores (Chirichella et al., 2021; Gaillard et al., 2015). In ungulates, this age‐related tooth wear is considered the major proximal cause of senescence due to the accompanying reduction of foraging ability and the associated loss of body condition (Carranza et al., 2004; Chirichella et al., 2021; Ericsson & Wallin, 2001; Pérez‐Barbería, 2019).

## 3 THE PERMANENT DENTITION OF THE EUROPEAN ROE DEER

The permanent dentition of the European roe deer typically comprises 32 teeth (Kierdorf & Kierdorf, 1989a). There are eight mandibular front teeth, namely, the six incisors plus the two incisiform canines that are located directly adjacent to the third incisors. The mandibular front teeth are separated from the cheek teeth by a wide diastema. As in ruminants in general, upper incisors are missing and the lower incisors and canines work against a horny pad in the premaxillary region. Upper canines are mostly also missing from the roe deer dentition. However, in some individuals, they can be present either uni‐ or bilaterally, and the frequency of their occurrence varies among populations (del Garcia Rincon et al., 2022; Kierdorf et al., 2007).

**FIGURE 1:**
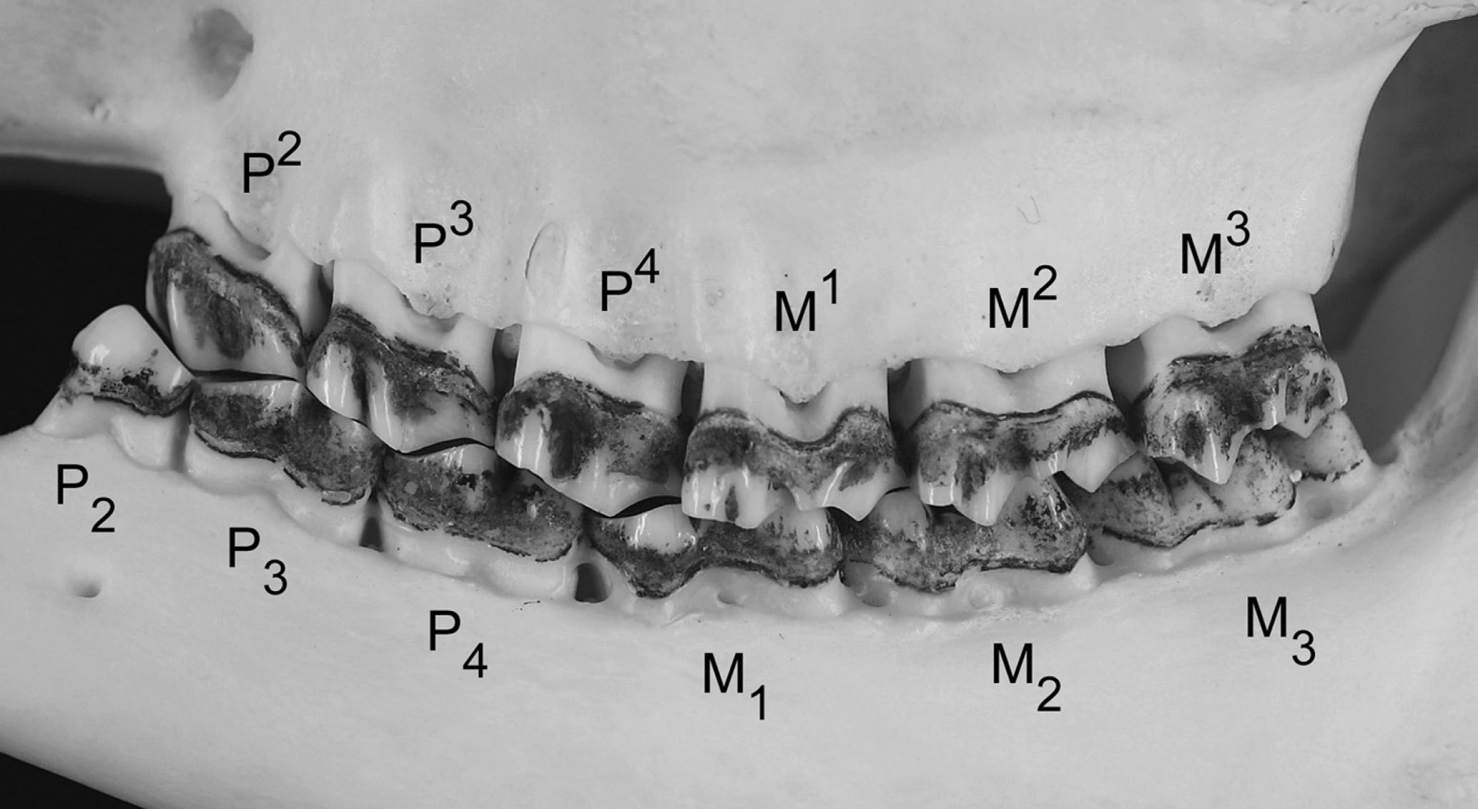
Buccal view of the left maxillary and mandibular permanent cheek tooth rows of a European roe deer. Note that except for the P_2_ and the M^3^, all teeth occlude with two antagonists.

The six cheek teeth present in each jaw quadrant are the second, third, and fourth permanent premolars (P2‐4) and the first, second, and third molars (M1‐3) (Figure 1). First premolars are absent in roe deer (except for rare cases, see Kierdorf, 1993) and other ruminants. Thus, the most mesial (anterior) cheek tooth is the second premolar. The crowns of the cheek teeth of roe deer (and other cervids) are lower and possess more prominent roots than those of bovids, and roe deer molars have been characterized as brachydont (Eidmann, 1939; Thenius, 1989). On the basis of cusp morphology (Fortelius, 1985; Hillson, 2005), the permanent cheek teeth of the roe deer can be classified as bunodont (P_2_), lophodont (P_3_), selenolophodont (P_4_, anterior lobe selenodont, posterior lobe lophodont), or selenodont (mandibular molars and all maxillary cheek teeth).

As the mandibular cheek teeth are positioned anteriorly relative to their maxillary homologs, except for the P_2_ and the M^3^, each permanent cheek tooth in the roe deer dentition occludes with two antagonists. Thus, most mandibular cheek teeth occlude with the mesial portion of their homolog in the maxilla and the distal (posterior) portion of the cheek tooth located anterior to it (Figure 1). The mesial part of the P_2_ is quite often not in contact with the P^2^ and therefore unworn or only slightly worn. The M_3_ is three‐lobed and occludes with the posterior lobe of the M^2^ and the two‐lobed M^3^ (Figure 1).

## 4 FLUORINE IN THE ENVIRONMENT

Fluorine is the most electronegative of all elements and ubiquitous in the environment. Due to its high reactivity, fluorine does not occur in the elemental state in nature but forms compounds with other elements, mostly in the form of minerals containing the fluoride anion (F^−^) (Fuge, 2019; Greenwood & Earnshaw, 1997). Fluorides are released into the biosphere by different natural processes, such as rock weathering, volcanic activity, and the formation of marine aerosols. In addition, fluorides in gaseous and particulate forms are discharged from various anthropogenic sources via exhaust fumes, process waters, and waste material. Human activities known to release larger amounts of fluorides into the environment are coal burning, the production of aluminum and other metals, the manufacture of bricks, glass, ceramics, and adhesives, and the production and use of phosphate fertilizers, fluoride‐containing pesticides, and high‐octane fuels (Fuge, 2019; Gutowska et al., 2019; Ranjan & Ranjan, 2015; WHO, 2002). The addition of fluoride to public drinking‐water supplies in some countries and the use of fluoride‐containing dental products further contribute to anthropogenic fluoride release (Fuge, 2019; WHO, 2002). Organofluorine compounds such as per‐ and polyfluoroalkyl substances (PFAS), which are also widely present in the environment (Diekman & Aga, 2022; Fuge, 2019; Peritore et al., 2023), are outside the scope of this review.

In the general public, fluoride is probably best known for its successful use in the control of dental caries (Buzalaf et al., 2011; Ellwood et al., 2008). However, as exposure to higher levels of fluoride, whether from natural or anthropogenic sources, has various negative effects on organisms (Gutowska et al., 2019; Ranjan & Ranjan, 2015; WHO, 2002; Wu et al., 2022), fluoride must also be considered a potential health hazard. Thus, for example, worldwide, the health of many million people, most of them residing in Asia or Africa, is negatively impacted by the consumption of groundwater with elevated concentrations of geogenic fluoride (Podgorski & Berg, 2022; Srivastava & Flora, 2020). The guideline value for the fluoride content of drinking water set by the World Health Organization is 1.5 mg/L (WHO, 2004, 2017), while, for example, in India, the desirable maximum fluoride concentration in drinking water is 1.0 mg/L (BIS, 2012). In regions with elevated fluoride concentrations in groundwater, not only humans but also their domestic animals are affected by chronic fluoride poisoning (Choubisa et al., 2023), and it has been asked whether wildlife species are also at risk (Choubisa, 2024a). In areas subject to the fallout of volcanic ash, fluoride toxicosis (including dental and skeletal fluorosis) in domestic and wild herbivores due to the uptake of fluoride‐rich tephra particles has repeatedly been observed (Cronin et al., 2003; Flueck, 2016; Flueck & Smith‐Flueck, 2013; Georgsson & Petursson, 1972; Roholm, 1937).

Indoor burning of coal with a high content of fluorine and other elements like arsenic and mercury in unvented stoves constitutes a further human health hazard, which in China alone affects millions of people (Finkelman et al., 1999; Guan, 2021). The emission of fluorides in gaseous and particulate forms from industrial sources, such as primary aluminum smelters and coal‐fired power plants, has been shown to negatively impact the environment and its biota at a local or regional level (Death et al., 2015, 2017; Jelenko & Pokorny, 2010; Kierdorf et al., 2012; Kierdorf & Kierdorf, 2002; Vikøren & Stuve, 1996). In our studies on roe deer, excess fluoride exposure of the animals resulted mainly from the burning of coal (mostly brown coal) in power plants, which caused fluoride pollution of larger areas around these emission sources (Kierdorf, 1988; Kierdorf et al., 1999, 2012; Zemek et al., 2007). As was shown for the formerly heavily polluted region of North Bohemia (Czech Republic), the installation of effective emission control devices in the power plants was followed by a pronounced decline in fluoride pollution of the study area and an associated marked drop in bone fluoride levels and dental fluorosis prevalence in resident deer (Kierdorf et al., 2012).

Depending on the ingested dose and the duration of exposure, uptake of excessive amounts of fluoride causes acute or chronic toxicosis in humans and other mammals, affecting both mineralized and non‐mineralized tissues (Gutowska et al., 2019; NRC, 1974; Ranjan & Ranjan, 2015; Shupe & Olson, 1983; Whitford, 1996). Susceptibility to chronically increased fluoride exposure varies among species, with, for example, sheep and goats considered to be able to tolerate somewhat higher dietary fluoride levels than cattle (Choubisa, 2024b; Ranjan & Ranjan, 2015). Within a given species, the outcome of increased fluoride uptake is affected by various factors, including age, sex, genetic background, overall health status, and physical activity, as well as the supply of calcium and vitamin D (Dey Bhowmik et al., 2021; Everett, 2011; Hu et al., 2024; Kobayashi et al., 2014; Ranjan & Ranjan, 2015; Simon et al., 2014; Whitford, 1996).

## 5 DENTAL FLUOROSIS—A MANIFESTATION OF CHRONIC FLUORIDE TOXICITY

Dental fluorosis is a developmental defect of enamel caused by prolonged ingestion of excess amounts of fluoride during odontogenesis (DenBesten & Li, 2011; Fejerskov et al., 1988; Roholm, 1937; Shupe & Olson, 1983). The condition is also referred to as enamel fluorosis or mottled enamel (Dean, 1936; DenBesten & Li, 2011; Everett, 2011). Excessive fluoride intake also causes pathological changes in the dentin, which forms the bulk of the teeth. Contrary to enamel, the fluorotic lesions in dentin cannot be assessed by external inspection but require radiographic or histological analysis (Appleton, 1994; Appleton et al., 2000; Fejerskov et al., 1979; Kierdorf et al., 1993; Okamoto et al., 2025; Richter et al., 2010; Rojas‐Sánchez et al., 2007; Walton & Eisenmann, 1975; Yaeger & Eisenmann, 1963). Elevated fluoride uptake after completion of crown formation in the permanent dentition does not result in dental fluorosis.

Mature dental enamel is a highly mineralized tissue (more strictly speaking, a cell‐free mineralized extracellular matrix) that forms the outer layer of the tooth crown and constitutes the hardest substance of the mammalian body (Boyde, 1989). Dental enamel is produced by specialized ectodermal cells, the ameloblasts, which are lost from the teeth upon their eruption into the oral cavity (Nanci, 2018). Contrary to bone tissue, enamel is therefore incapable of remodeling or cell‐mediated repair. In consequence, fluoride‐induced enamel changes are permanent and constitute a lifelong biomarker of excessive fluoride intake during dental development (DenBesten & Li, 2011; Fejerskov et al., 1988; Kierdorf & Kierdorf, 1999).

Enamel formation (amelogenesis) is divided into different stages, namely, a pre‐secretory stage, a secretory stage, a short transitional stage, and finally a maturation stage (Nanci, 2018). During the secretory stage, a protein matrix is secreted by the ameloblasts in a cyclically modulated fashion and undergoes initial mineralization immediately after its secretion (Gil‐Bona & Bidlack, 2020; Nanci, 2018; Wu et al., 2024). Unlike in bone or dentin, there is therefore no rim of unmineralized matrix at the forming front of enamel. The periodic oscillation in ameloblast activity during the secretory stage is reflected by the presence of regular incremental markings in enamel (Boyde, 1989; Emken et al., 2021; Kierdorf et al., 2019; Risnes, 1998; Smith, 2006; Wu et al., 2024).

The bulk of the enamel matrix consists of amelogenins, which in humans and several other mammalian species are encoded by two genes located, respectively, on the X chromosome (*AMELX*) and the Y chromosome (*AMELY*), while rodent species possess only *AMELX* (Gil‐Bona & Bidlack, 2020). In addition to being encoded by two different genes in many species, the alternative splicing of their mRNA contributes to the heterogeneity of the amelogenins. One function of amelogenins is to inhibit the lateral growth of the enamel crystals (Nanci, 2018). Other structural enamel matrix proteins are ameloblastin and enamelin, which, very probably due to rapid extracellular processing, are present in much smaller amounts than amelogenins (Nanci, 2018). The enamel matrix also contains enzymes (enamelysin (matrix‐metalloproteinase‐20, MMP20) and kallikrein‐4 (KLK4)) that function in the post‐secretory processing and degradation of the matrix proteins (Gil‐Bona & Bidlack, 2020; Nanci, 2018).

During the maturation stage, which starts after the full thickness of (immature) enamel has been laid down, the enamel matrix is enzymatically broken down, and the degraded matrix constituents and water are removed. The resulting spaces are filled in by the growth of the enamel crystals to their final size following the massive influx of mineral ions (Gil‐Bona & Bidlack, 2020; Kallistová et al., 2017; Nanci, 2018; Smith, 1998). Maturation starts at the crown tips, and the degree of mineralization gradually increases from cuspal to cervical and from inner to outer enamel (Green et al., 2017; Smith, 1998). When the maturation process is completed, the enamel consists of about 96 to 98% mineral (bioapatite) by weight, and its crystals are so tightly packed that it appears translucent (Boyde, 1989).

The pathological features of fluorotic enamel have been intensely studied in humans and other mammalian species (Boulton et al., 1997; Bronckers et al., 2009; Fejerskov et al., 1974, 1975, 1977; Kierdorf et al., 1993, 1996, 1997, 2004, 2016; Shupe & Olson, 1983; Suckling et al., 1988; Thylstrup & Fejerskov, 1978), and different hypotheses regarding the pathophysiological mechanism of dental fluorosis have been proposed (Aulestia et al., 2020; Bronckers et al., 2009; DenBesten & Thariani, 1992; Everett, 2011; Fejerskov et al., 1977, 1994; Jalali et al., 2017; Johnston & Strobel, 2020; Sharma et al., 2010). Excess fluoride impairs the secretory and maturation stages of amelogenesis, with the pathological changes depending on the intensity and timing of fluoride exposure, the resulting (constant or fluctuating) plasma fluoride levels, and the stage within the life span of the affected ameloblasts (Bronckers et al., 2009; DenBesten & Thariani, 1992; Kierdorf et al., 2004).

## 6 FEATURES OF FLUOROTIC ENAMEL AND DIFFERENTIAL DIAGNOSIS OF DENTAL FLUOROSIS

**FIGURE 2:**
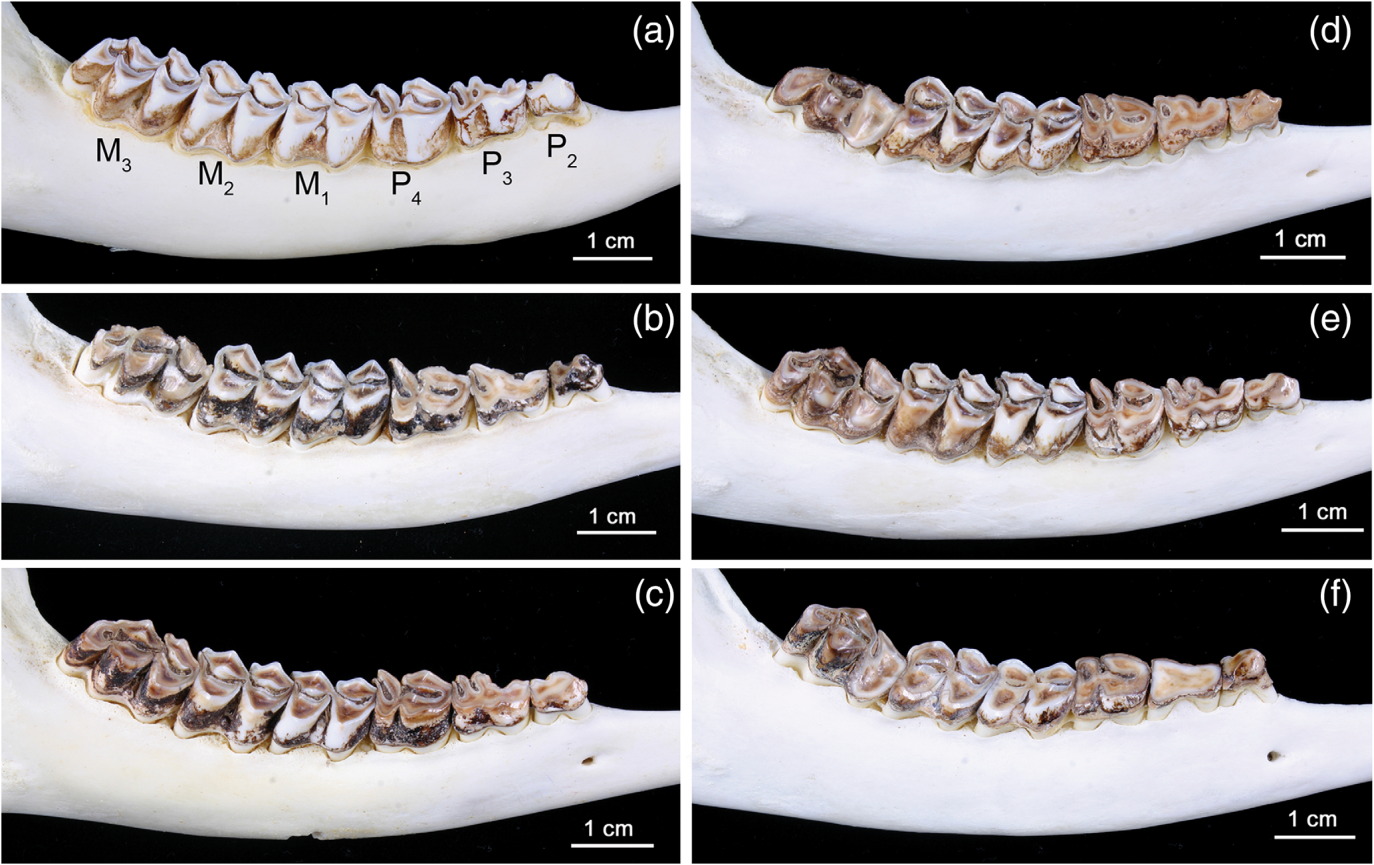
Right mandibular cheek tooth rows (bucco‐occlusal views) of adult male European roe deer collected between 1992 and 1997 in different areas of North Bohemia, Czech Republic. (a) Tooth row free of dental fluorosis with all teeth showing normal (whitish and glossy) enamel. (b–f) Fluorotic tooth rows exhibiting the characteristic distribution of dental fluorosis in this species. The three premolars (P_2‐4_) and the third molar (M_3_) show opacity and brownish staining of the enamel and intensified wear, particularly pronounced in the anterior (mesial) lobe of the M_3_. Height reduction is particularly prominent in the mesiobuccal cusp (protoconid) of this tooth. Note the normal appearance of the M_1_ and only slight fluorotic changes in the M_2_. Fluoride concentrations (mg/kg dry wt) in mandibular bone: (a) 974, (b) 2965, (c) 1514, (d) 1789, (e) 2030, (f) 3900.

**FIGURE 3:**
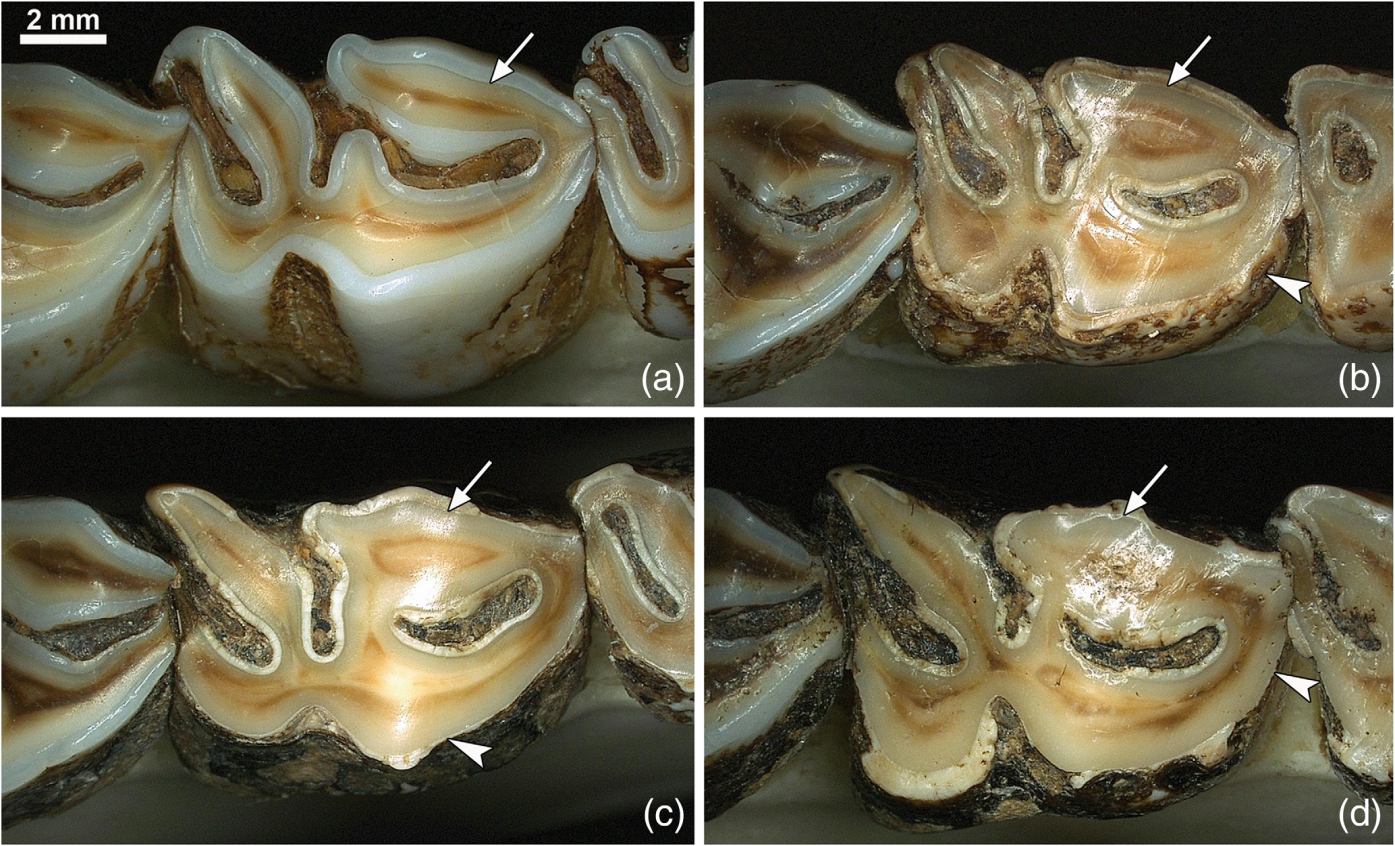
Occlusal views of right mandibular fourth premolars of male European roe deer with normal (a) or fluorotic (b–d) enamel; anterior (mesial) to the right of the images. (a) In the control P_4_, the enamel is of normal thickness and shows the typical whitish and glossy appearance. The enamel forms distinct ridges on the occlusal surface that protrude above the yellowish/brownish dentin. Arrow: EDJ. (b–d) The fluorotic enamel is opaque and shows a brownish staining. It does not form ridges on the occlusal surface, which is abnormally flat. The teeth show extended defects (arrowheads) where larger parts of enamel have flaked off posteruptively, in places leading to a considerable thinning of the enamel layer. Arrows: EDJ. Note the normal appearance of the enamel in the M_1_ (to the left of the P_4_) of the fluorotic dentitions (b–d). The scale bar in (a) also applies to the other panels.

**FIGURE 4:**
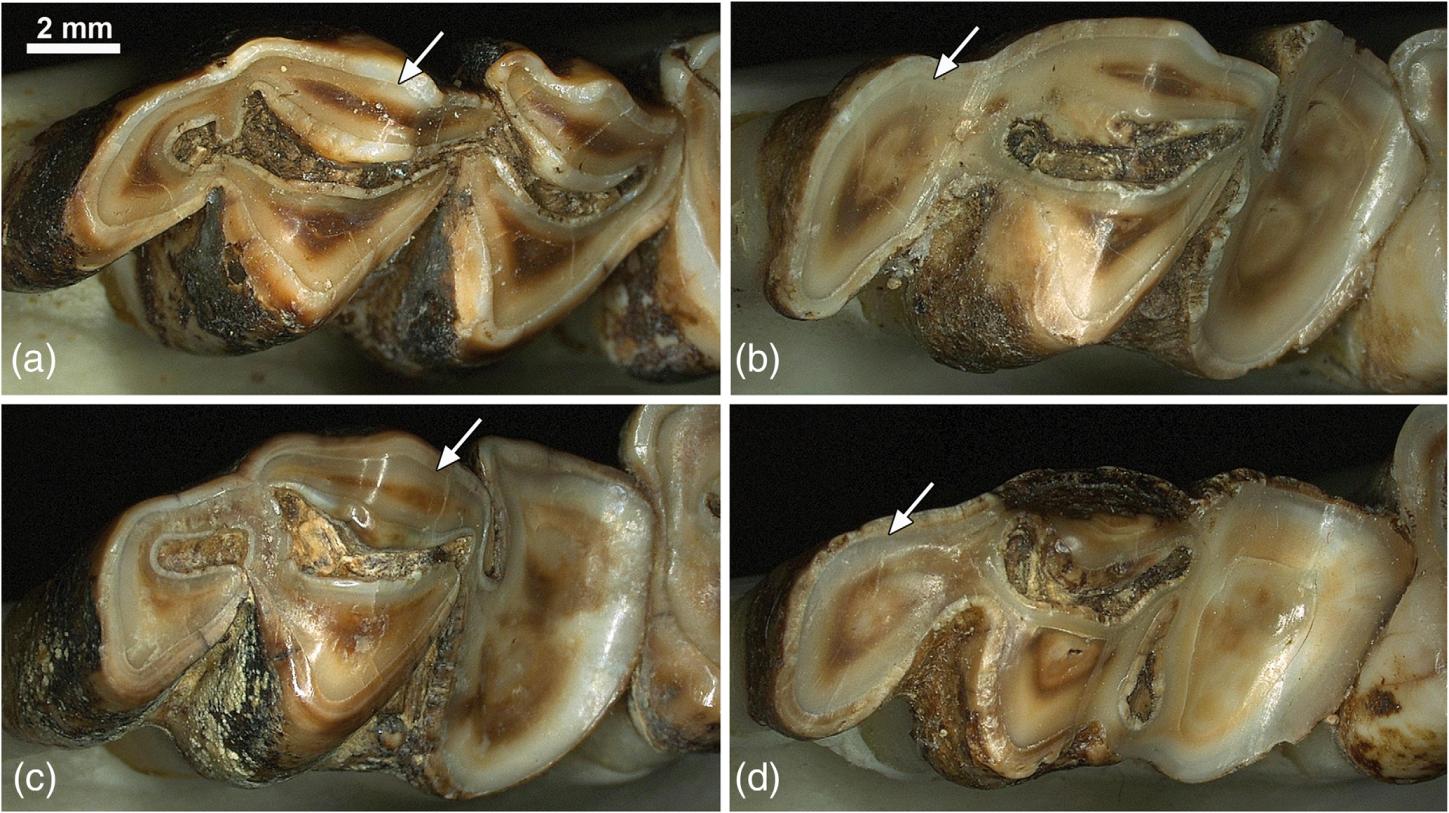
Occlusal views of fluorotic right M_3_ of four male European roe deer; anterior (mesial) to the right of the images. The enamel is opaque and stained, does not form ridges on the occlusal surface, and shows post‐eruptive lesions. Note abnormal wear of the teeth that is especially pronounced in the mesial lobe. Arrows: EDJ. The scale bar in (a) also applies to the other panels.

**FIGURE 5:**
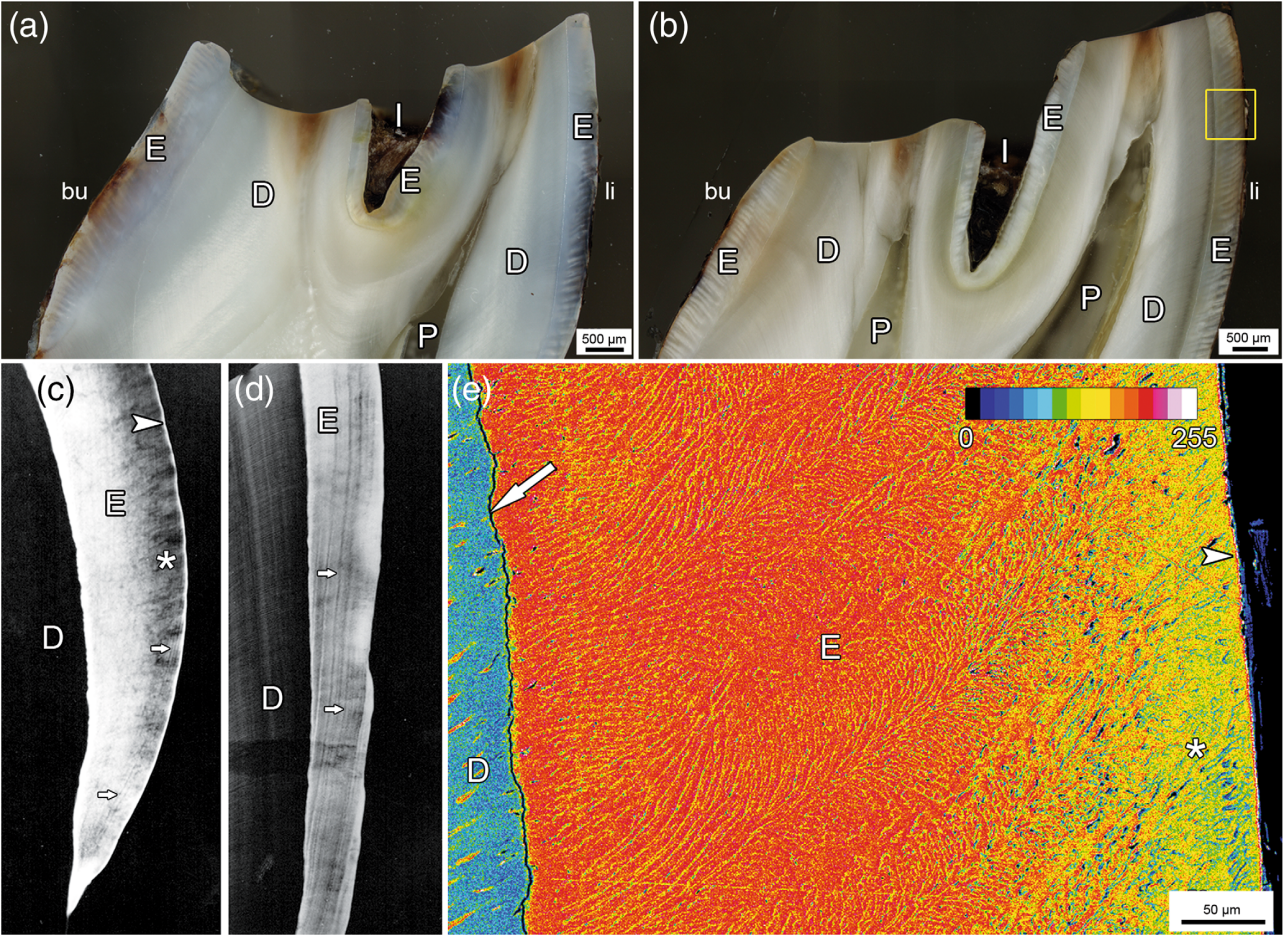
Appearance of enamel in non‐fluorotic (a) and fluorotic (b–e) enamel of permanent cheek teeth of European roe deer. (a, b) Reflected light images of polished block surfaces of two axiobuccolingually sectioned molars from the same individual. The sections run through the anterior (mesial) lobes of an M_1_ (a) and an M_3_ (b), the left cusp is the protoconid, and the right cusp is the metaconid. Note the presence of distinct enamel ridges on the occlusal surface of the M_1_ and the lack of such ridges in the M_3_, whose occlusal surface is abnormally flat. The yellow rectangle in (b) indicates the area shown in (e). D, dentin; E, enamel; I, infundibulum; P, pulp cavity; bu, buccal; li, lingual. (c, d) Microradiographs of fluorotic enamel of axiobuccolingual ground sections through fluorotic roe deer cheek teeth. D, dentin; E, enamel. (c) Note hypomineralization of outer enamel (asterisk) underneath a thin, more highly mineralized surface rim (arrowhead) and an increase in the degree of enamel mineralization from the subsurface layer toward the EDJ. Also note alternating layers of higher and lower mineral content whose orientation corresponds to that of incremental lines (arrows). (d) In this specimen, the pattern of alternating lines with higher and lower mineral content is more pronounced (arrows), and the hypomineralization extends throughout the entire enamel. (e) Scanning electron micrograph (backscattered electron mode) of the area of fluorotic enamel indicated by the yellow rectangle in (b). For visualization, the 256 gray values from black (0) to peak white (255) were converted to 16 colors as shown in the inset, with higher gray values (brighter gray levels) indicating higher degrees of mineralization. A pronounced hypomineralization of the outer enamel (asterisk) underneath a thin outermost rim of higher mineral content (arrowhead) and an increase in mineralization from the subsurface zone toward the EDJ is well visible. D, dentin; E, enamel; arrow, artefactual cleft denoting the position of the EDJ.

The most conspicuous macroscopic feature of fluorotic enamel in roe deer (Kierdorf, 1988; Kierdorf et al., 1993) and other mammalian species (Bronckers et al., 2009; Death et al., 2015; Fejerskov et al., 1977, 1988; Kierdorf et al., 1996, 2000, 2004, 2016; Shupe & Olson, 1983; Suckling et al., 1988; Thylstrup & Fejerskov, 1978) is its opaque (sometimes chalky) appearance and posteruptive brownish staining (Figures 2, 3, 4). The opacity results from disturbed mineralization that leads to abnormally porous enamel exhibiting extended periprismatic gaps and increased intercrystalline spaces (Fejerskov et al., 1974, 1977). The hypomineralization of fluorotic roe deer enamel has been demonstrated by hardness testing (Kierdorf, 1988), microradiography (Kierdorf et al., 1993), and backscattered electron imaging (Kierdorf et al., 1997) (Figure 5). Hypomineralization of fluorotic mammalian enamel is often most pronounced in the outer zone underneath a thin, more highly mineralized surface layer, and the mineral content of fluorotic enamel increases from the hypomineralized outer zone toward the enamel–dentin junction (EDJ) (Figure 5c,d) (Fejerskov et al., 1975, 1977; Kierdorf et al., 1993, 1996, 1997, 2016; Richards et al., 1992). For fluorotic roe deer enamel, this situation is shown in Figure 5c,e; however, as demonstrated by Figure 5d, other patterns of hypomineralization can also be found. It has been shown that the surface rim of higher mineral content develops prior to tooth eruption (Lyaruu et al., 2014; Richards et al., 1992). Upon tooth eruption, fluorotic enamel is whitish‐opaque. The characteristic staining of the fluorotic enamel forms posteruptively and is attributed to infiltration of substances from the oral cavity into the porous tissue (Kierdorf, 1988; Kierdorf et al., 1993).

The protein content of fluorotic enamel exceeds that of healthy enamel. The retention of amelogenins is suggestive of a reduced proteolytic activity in the fluorotic enamel matrix during the maturation stage (DenBesten & Li, 2011; Gil‐Bona & Bidlack, 2020). It has been suggested that, in the presence of high fluoride levels, the massive precipitation of apatite during the maturation stage and the associated drop in matrix pH cause the formation of large amounts of HF (Sharma et al., 2010). Compared to the fluoride anion (F^−^), HF can much more easily diffuse across the cell membrane, which is followed by dissociation into H^+^ and F^−^ in the neutral cytosol. The resulting high intracellular F^−^ level would then exert a toxic effect on the ameloblasts, leading to reduced release of matrix‐degrading proteins, increased protein retention within, and, finally, hypomineralization of the fluorotic enamel.

That the hypomineralization of fluorotic enamel basically reflects impaired enamel maturation was already proposed by Fejerskov et al. (1977). In line with this view, experimental studies in pigs (Richards et al., 1986) and sheep (Suckling et al., 1988) showed that the typical hypomineralization of fluorotic enamel can be induced when fluoride only impacts the maturation stage of amelogenesis. However, in the sheep study by Suckling et al. (1988), enamel hypomineralization increased in depth and severity when fluoride also affected the secretory stage. Notwithstanding the potential presence of such a carry‐over effect from the secretory stage, the typical subsurface hypomineralization of fluorotic enamel is regarded to basically reflect chronically increased fluoride exposure during the maturation stage (Bronckers et al., 2009; Sharma et al., 2010).

Fluorotic roe deer enamel frequently shows a distinct pattern of alternating lines/bands of higher and lower mineral content, whose orientation matches that of the incremental lines formed during the secretory stage (Figure 5c,d) (Kierdorf et al., 1993). This feature has also been observed in fluorotic enamel of red deer (Kierdorf et al., 1996) and pigs (Kierdorf et al., 2004). It could be indicative of a fluoride impact on the initial mineralization during the secretory stage of amelogenesis, the effects of which persist through enamel maturation. The formation of a highly mineralized surface rim and of hypermineralized lines deeper within the enamel was observed in (permanently growing) incisors of mice given drinking water with 100 mg fluoride/L (Lyaruu et al., 2014). These authors suggested that excess fluoride stimulated repeated instances of hypermineralization at the respective mineralization front during the secretory stage and that the resulting hypermineralized lines formed barriers to the transport of mineral ions and enzymes into the underlying enamel, causing the formation of hypomineralized layers. The proposed mechanism could, in principle, also explain the pattern of alternating lines with higher and lower mineral content observed in fluorotic roe deer enamel.

**FIGURE 6:**
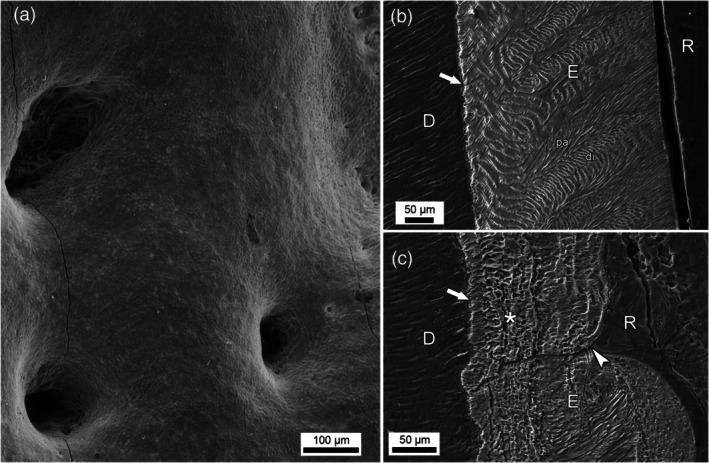
Hypoplastic defects of fluorotic roe deer enamel and associated structural abnormalities of the enamel. (a) Scanning electron micrograph (secondary electron mode) of the buccal enamel surface of a freshly erupted fluorotic P_4_ exhibiting narrow and deep hypoplastic defects. Part of a more extended and shallower hypoplastic defect is visible in the upper right corner of the image. Note that the rounded walls of the defects are covered by numerous small depressions (Tomes' process pits) that characterize intact enamel surfaces. (b) Scanning electron micrograph (secondary electron mode) of buccal enamel of an axiobuccolingually sectioned, resin‐embedded M_1_ from a fluorotic dentition. The polished section surface was etched with 34% phosphoric acid for 5 s. The enamel shows a normal microstructure with Hunter–Schreger bands in deeper enamel, consisting of alternating zones with more transversely (diazones, di) and more longitudinally sectioned (parazones, pa) prisms. Closer to the enamel surface, the prisms are all more or less arranged in parallel. D, dentin; E, enamel; R, resin; arrow, EDJ. There is an artefactual cleft between the resin and enamel surface. (c) Scanning electron micrograph (secondary electron mode) of etched (34% phosphoric acid for 5 s) buccal enamel of an axiobuccolingually sectioned, resin‐embedded M_3_ from the same fluorotic dentition as the M_1_ shown in (b). Note the presence of a hypoplastic defect with rounded walls (arrowhead) and disturbed enamel structure with layers of prismless enamel (asterisk). D, dentin; E, enamel; R, resin; arrow, EDJ.

A further pathological feature of fluorotic roe deer teeth is the presence of enamel hypoplasia (Figure 6a,c). These developmental defects denote a fluoride impact on the secretory ameloblasts that led to a temporary or permanent reduction or stop of enamel matrix secretion (Kierdorf et al., 1993; Kierdorf & Kierdorf, 1989b, 1997). Size and shape of the hypoplastic defects vary widely, and in some teeth the co‐occurrence of deep and narrow (funnel‐type) pits and more extended and shallower defects has been observed (Figure 6a). On external inspection, enamel hypoplasia can be distinguished from lesions caused by posteruptive loss of surface enamel based on the rounded margins of the hypoplastic defects and the fact that their walls are covered by Tomes' process pits, which are a characteristic feature of intact enamel surfaces (Figure 6a).

Hypoplastic pitting has also been observed in fluorotic enamel of sheep (Purdell‐Lewis et al., 1987; Suckling & Thurley, 1984), red deer (Kierdorf et al., 1996; Kierdorf & Kierdorf, 1997), wild boar and domestic pigs (Kierdorf et al., 2000, 2004), and eastern gray kangaroos (Kierdorf et al., 2016). In the incisor enamel of sheep, hypoplastic pits were experimentally produced by administering daily oral doses of 2, 4, or 6 mg fluoride (given as NaF) per kg body weight for 21 days (Purdell‐Lewis et al., 1987) or of 4 mg fluoride/kg body weight for 26 days (Suckling & Thurley, 1984). Pit‐type hypoplastic defects were also observed in the enamel of mandibular third molars of domestic pigs receiving a daily oral dose of 0.9 mg fluoride (given as NaF) over a period of 1 year (Kierdorf et al., 2004). The treatment period covered most of the secretory stage and the entire maturation stage of amelogenesis of this tooth.

In rodents, enamel hypoplasia has been produced by parenteral administration of high doses of fluoride. Thus, subcutaneous (single dose) injections of 30 mg NaF/kg body weight in rats (Lange Nordlund et al., 1986) and of 40 mg NaF/kg body weight in hamsters (Lyaruu et al., 2012) led to hypoplastic pitting of the enamel that was associated with the formation of so‐called sub‐ameloblastic cysts due to the detachment of the secretory ameloblasts from the surface of the forming enamel. Structural changes of enamel suggestive of a local temporary detachment of the ameloblast layer from the surface of the forming enamel have also been observed in wild boar (Kierdorf et al., 2000) and domestic pigs (Kierdorf et al., 2004) exposed to excess fluoride.

The occurrence of hypoplastic defects in the fluorotic enamel of roe deer and other mammals is associated with the presence of prismless enamel (Kierdorf et al., 1993, 1996, 2000, 2004, 2016; Kierdorf & Kierdorf, 1997). Figure 6b shows the normal structure of roe deer enamel as seen in the SEM, while Figure 6c depicts the disturbed microstructure of fluorotic enamel. Formation of prismless enamel denotes a markedly reduced activity of secretory‐stage ameloblasts that goes along with a reduction of the distal, prism‐forming portion of their Tomes' process. This results in the presence of a single, more or less flat surface at the secretory pole of the cell. The crystals forming in the matrix secreted at this surface are arranged more or less in parallel and show the orientation characteristic of interprismatic enamel (Kierdorf et al., 1993; Kierdorf & Kierdorf, 1997).

The differential diagnosis of dental fluorosis, that is, the distinction of this condition from pathological enamel changes not related to fluoride, is a matter of some debate. Regarding the clinical diagnosis of dental fluorosis in humans it has been concluded that “enamel changes resulting from long‐term ingestion of fluoride during the period of tooth development are very distinctive” and that “it is most unlikely that any enamel changes of non‐fluoride origin will constitute a differential diagnostic problem” (Fejerskov et al., 1988, p. 62). This view is supported by Pendrys (1999, p. 238) who states that “existing evidence supports the validity of differential clinical diagnosis of enamel fluorosis when appropriate criteria are employed.” In contrast, Cutress and Suckling (1990) emphasize the risk of misdiagnosing enamel changes as fluoride‐induced and call for a more discriminating diagnostic procedure that includes the history of fluoride exposure of individuals. More recently, it has been concluded that it is possible to distinguish dental fluorosis from the enamel defect known as molar incisor hypomineralization on the basis of chemical and structural analyses at different levels of resolution (Gil‐Bona & Bidlack, 2020; Houari et al., 2023).

Fluoride concentrations in blood, urine, saliva, hair, nails, dental hard tissues, or bones of individuals are widely used as biological markers of fluoride exposure in humans (Duckworth et al., 2021; Pessan & Buzalaf, 2011; Rugg‐Gunn et al., 2011). Data on fluoride levels in bone, dentin, and enamel were also collected in several of our studies on roe deer to substantiate an increased fluoride exposure of the animals and thereby support the diagnosis of dental fluorosis (Kierdorf, 1988; Kierdorf et al., 1991, 2012; Richter et al., 2011).

## 7 SEQUELAE OF DENTAL FLUOROSIS

**FIGURE 7:**
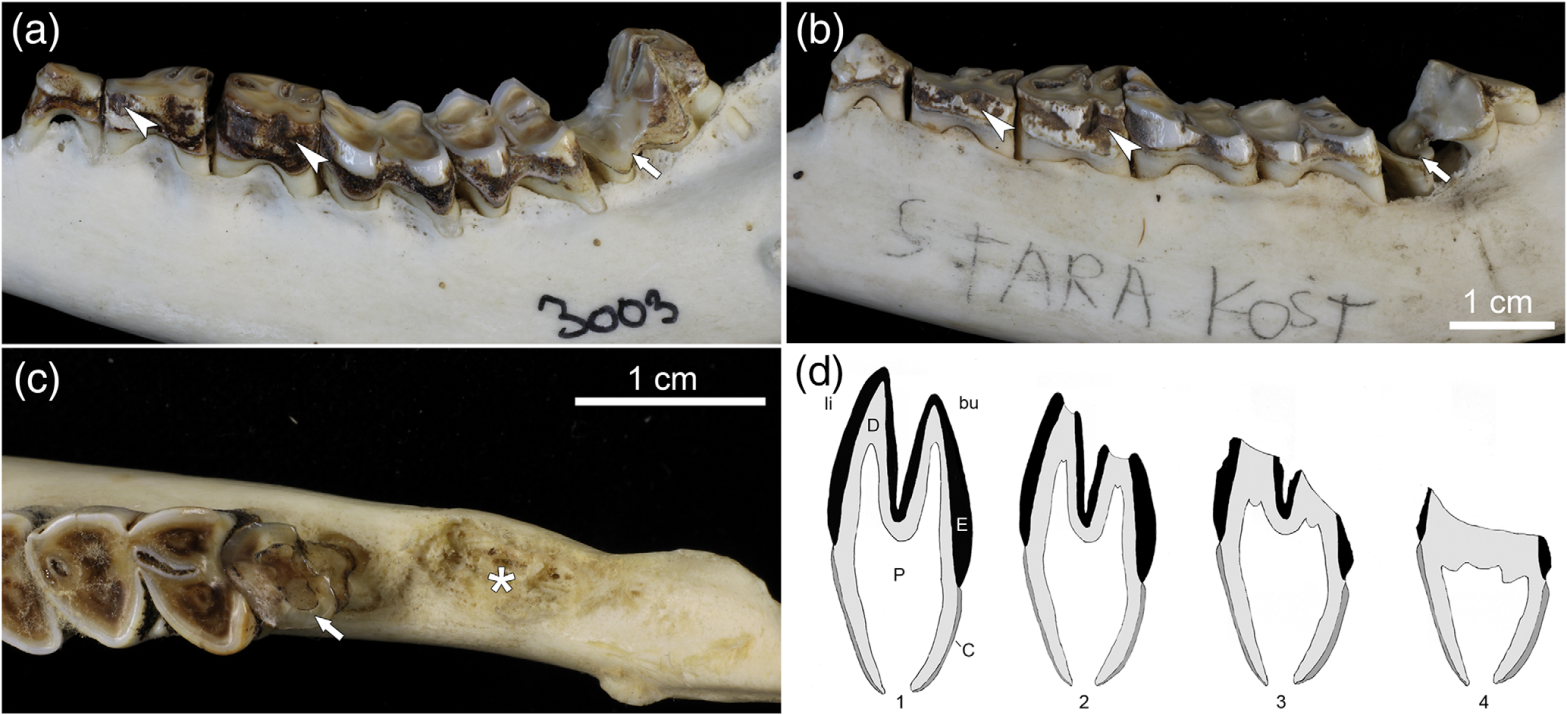
Pathological changes in the M_3_ of fluorotic mandibular cheek tooth rows of European roe deer. (a) Buccal view of fluorotic cheek tooth row of a roe deer from the Czech Republic. Note the grossly increased wear of the anterior (mesial) portion of the M_3_ (arrow). The crown of the tooth has been worn away in this area, and the wear surface is located in the roots. Arrowheads: posteruptive enamel lesions in P_3_ and P_4_. The scale bar in (b) also applies to (a). (b) Buccal view of fluorotic cheek tooth row of a roe deer from Slovenia. The grossly increased wear of the anterior (mesial) portion of the M_3_ is associated with height reduction of the alveolar bone and has almost caused a separation of the anterior portion from the rest of the tooth. Arrowheads: posteruptive enamel lesions in P_3_ and P_4_. (c) Occlusal view of posterior (distal) portion of a fluorotic cheek tooth row of a roe deer from Germany. Except for a small, anterior remnant (arrow), the M_3_ has been lost from the mandible due to severe periodontal breakdown, and the alveolus has become filled with new bone (asterisk). (d) Schematic illustration of transverse sections through the anterior (mesial) lobe of roe deer M_3_ exhibiting normal and abnormal wear patterns. 1, freshly erupted, unworn tooth with cusp tips still covered by enamel; 2, normal wear pattern with presence of enamel ridges on the occlusal surface; 3, abnormal wear pattern of fluorotic tooth, no enamel ridges on occlusal surface, particularly severe wear on the protoconid; 4, situation after the wear surface has progressed beneath the level of the infundibulum, which occurs prematurely in fluorotic teeth. C, cementum covering the tooth root; D, dentin; E, enamel; P, pulp cavity; bu, buccal; li, lingual.

In addition to posteruptive staining, the hypomineralization of fluorotic enamel in the European roe deer (Kierdorf, 1988; Kierdorf et al., 1993; Kierdorf & Kierdorf, 2000; Vikøren & Stuve, 1996) and other cervid species (Flueck & Smith‐Flueck, 2013; Garrott et al., 2002; Kierdorf et al., 1996; Shupe et al., 1984; Vikøren & Stuve, 1996) causes further secondary changes in erupted teeth. The most pronounced is an accelerated and abnormal wear of the teeth that includes the reduction in height or complete loss of the enamel ridges on the occlusal surface of the cheek teeth (Figures 3, 4, 5, and 7c) and often causes a premature loss of tooth function (Garrott et al., 2002; Kierdorf, 1988; Kierdorf et al., 1993, 1996; Vikøren & Stuve, 1996). Among the mandibular cheek teeth, M_3_ is typically affected the most, with its mesial (anterior) lobe being worn down particularly rapidly (Figures 2, 4, and 7a,b,d). As a further sequela of the intense wear of fluorotic teeth, periodontal breakdown with loss of alveolar bone may be observed in older individuals (Figure 7b,c). In particularly severe cases, this can cause tooth loss, especially of the M_3_ (Figure 7c).

Shupe and Olson (1983) have described an increased coarseness of the rumen content in cattle with severe dental fluorosis due to impaired mastication. This will increase ruminal retention time of the ingesta and reduce energy extraction from the diet, with negative impacts on the animals' physical condition. In wapiti from the Yellowstone National Park (USA) exposed to excess fluoride from geogenic sources, premature loss of function of fluorotic teeth caused early onset of senescence and pronounced reduction of life expectancy in affected individuals (Garrott et al., 2002). A reduced life expectancy had previously already been assumed for severely fluorotic red deer from the Czech Republic (Schultz et al., 1998).

**FIGURE 8:**
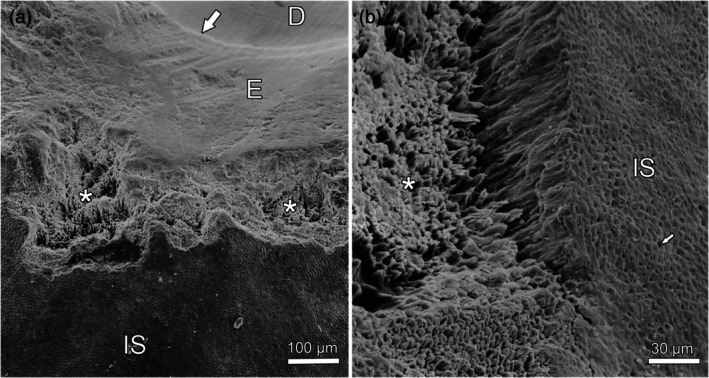
Scanning electron micrographs (secondary electron mode) showing posteruptive defects in fluorotic enamel of a roe deer M_3_. (a) Bucco‐occlusal view showing part of the occlusal surface. Larger portions of outer enamel have fractured away, causing the formation of extended defects with a ragged structure (asterisks). Note that the enamel does not form a ridge at the occlusal surface and shows deep scratches indicative of its reduced hardness. The intact buccal crown surface (IS) is densely covered by Tomes' process pits. D, dentin; E, enamel; arrow, EDJ. (b) Higher magnification of a defect area (asterisk) where outer enamel has been lost following fracture of the enamel prisms. The intact enamel surface (IS) is densely covered by Tomes' process pits (arrow).

A further feature of severely fluorotic deer teeth is enamel chipping, which demonstrates that the hypomineralized fluorotic enamel is unable to resist the mechanical forces acting upon it during mastication (Kierdorf et al., 1993, 1996; Kierdorf & Kierdorf, 1989b). Chipping is often prominent along the occlusal surface of the fluorotic teeth (Figure 8a). The bottom of the lesions exhibits a honeycomb‐like structure, with holes where individual prisms have broken away and a surrounding rim of interprismatic enamel (Figure 8b). Lesions caused by posteruptive chipping can easily be distinguished from hypoplastic defects of enamel (Kierdorf et al., 1993, 1996; Kierdorf & Kierdorf, 1989b).

## 8 VARIATION OF DENTAL FLUOROSIS SEVERITY AMONG THE PERMANENT CHEEK TEETH OF ROE DEER

As shown in Figures 2 and 7a,b, the intensity of fluorotic changes is not uniform among the permanent cheek teeth of roe deer. Rather, there exists a characteristic variation in the severity of dental fluorosis within the tooth row. This variation was most often studied in the mandibular premolars and molars because the mandibles are regularly collected by hunters for age estimation (Habermehl, 1985; von Raesfeld et al., 1978). Non‐fluorotic cheek teeth exhibit normal (whitish and glossy) enamel and a regular occlusal morphology (Figure 2a). The M_1_ is typically the most worn tooth as it erupts and comes into wear first. In contrast, in fluorotic roe deer mandibles, only the M_1_ shows normal enamel (very rarely minor fluorotic changes) and wear, while the enamel of the permanent premolars (P_2‐4_) and the third molar (M_3_) is opaque and stained (Figures 2b–f and 7a,b). Moreover, P_2–4_ and M_3_ show abnormal wear (Figures 3, 4, 5 and 7). Reduction of crown height is most pronounced in the mesial lobe of the M_3_, particularly its mesiobuccal cusp (protoconid) (Figures 4 and 7). The progressive breakdown of this tooth lobe in a fluorotic M_3_ is schematically illustrated in Figure 7d. Compared to P_2–4_ and M_3_, the second molar (M_2_) is less affected by fluorotic changes and typically shows more or less normal wear (Figures 2 and 7a,b).

**FIGURE 9:**
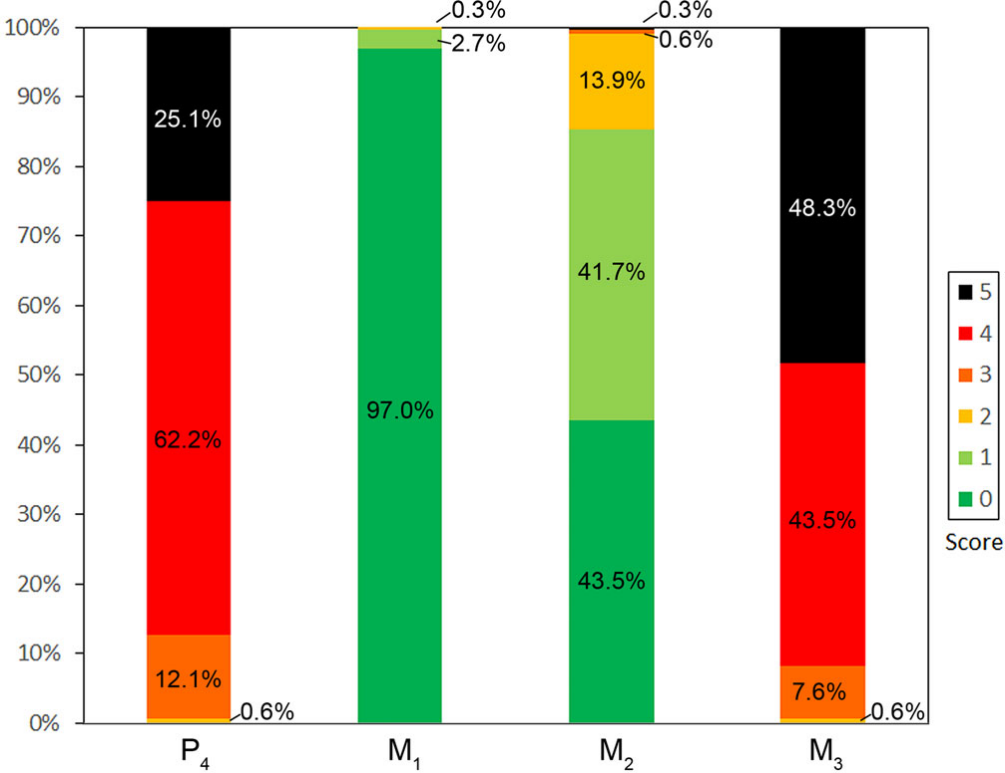
Scoring of the severity of dental fluorosis in P_4_ and M_1‐3_ from severely fluorotic (at least one tooth scoring 4 or 5) complete cheek tooth rows of European roe deer (*n* = 331) from the Czech–German border region (data from Kierdorf & Kierdorf, 2000). Score 0 = unaffected, scores 1–5 = increasing severity of fluorotic changes. Note that the M_1_ is almost always unaffected by dental fluorosis, whereas the P_4_ and M_3_ mostly exhibit a score of 4 or 5. The M_2_ is less frequently (43.5% with score 0) and less intensely affected by dental fluorosis than P_4_ and M_3_.

In previous studies, a scoring system for fluorotic changes in the permanent mandibular cheek teeth of deer (score 0: normal (non‐fluorotic) tooth; scores 1–5: increasing severity of pathological alterations (Kierdorf et al., 2012; Kierdorf & Kierdorf, 2000)) has been used to compare interdental variation in fluorosis severity. Figure 9 shows the distribution of the fluorosis scores for the P_4_ (representative of the permanent premolars) and the three molars (M_1‐3_) of 331 roe deer mandibles collected between 1992 and 1998 in the fluoride‐polluted Czech–German border region (Kierdorf & Kierdorf, 2000). All cheek tooth rows included were complete and exhibited severe dental fluorosis, that is, at least one of the six teeth exhibited a score of 4 or 5. The result highlights the regularity of interdental variation in fluorosis severity among the permanent mandibular cheek teeth of roe deer (cf. Figure 2).

**FIGURE 10:**
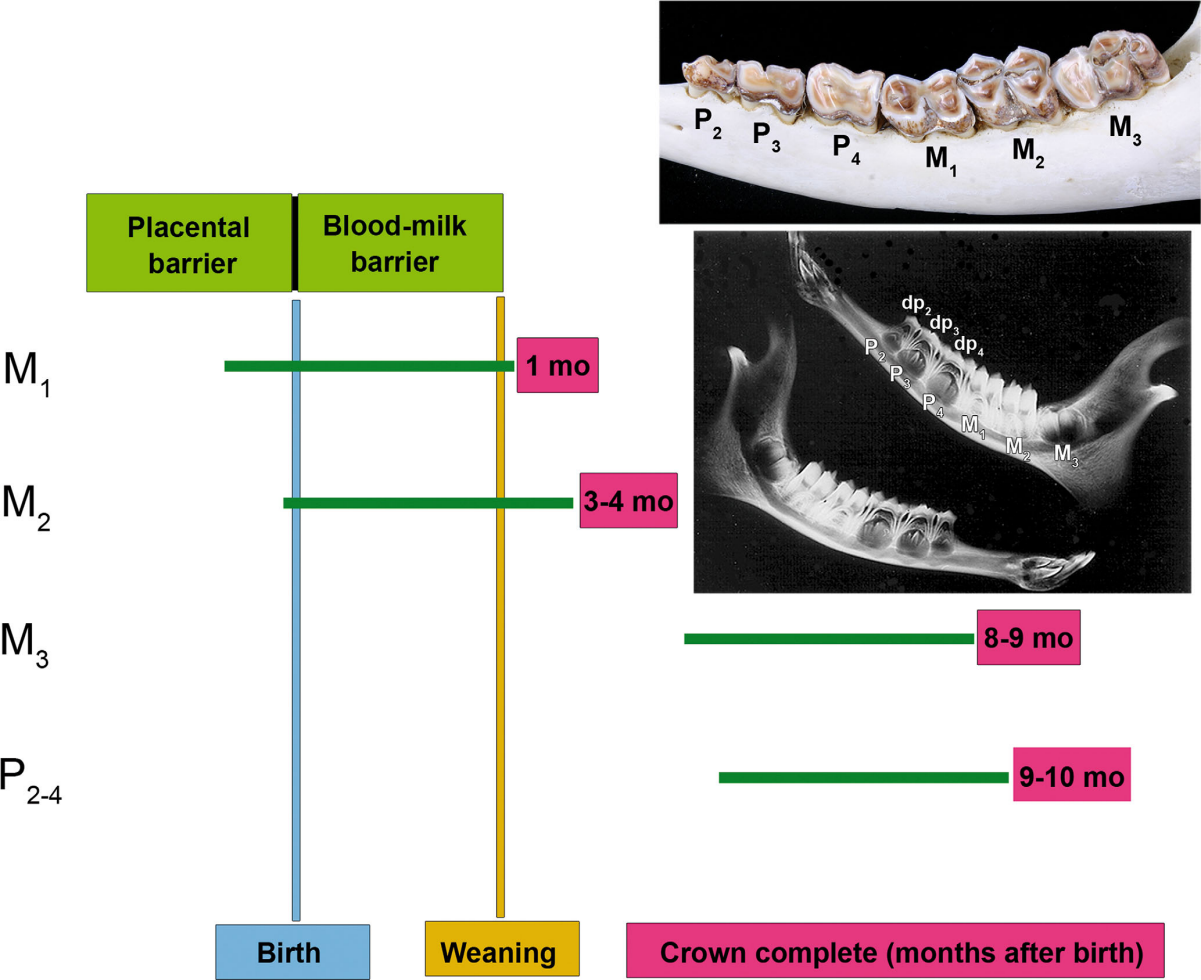
Schematic illustration of the approximate crown formation periods (green horizontal bars) for the permanent mandibular cheek teeth of the European roe deer in relation to birth and weaning, and of the approximate postnatal ages at crown completion for these teeth. The two protective mechanisms (placental barrier to fluoride and blood–milk barrier) supposed to operate during certain ontogenetic periods are indicated. The upper inset shows the typical distribution of dental fluorosis in the permanent mandibular cheek teeth of the European roe deer; the radiograph shows the stage of dental development in an approximately six‐month‐old individual. The deciduous premolars (dp), their still unerupted permanent successors (P), and the three molars (M) are indicated. Of the latter, M_1_ and M_2_ are fully erupted, while M_3_ is still in the crown formation stage.

The variation in fluorosis severity among its permanent mandibular cheek teeth has been related to dental development in the European roe deer (Kierdorf, 1988; Kierdorf & Kierdorf, 2000). This is schematically illustrated in Figure 10. Crown formation of the M_1_ starts prenatally and is completed shortly after weaning (Kierdorf & Kierdorf, 1989a). Crown formation in the M_2_ starts shortly before birth and is finished at about 3–4 months of age (Kierdorf & Kierdorf, 1989a). In contrast to M_1_ and M_2_, crown formation of the permanent premolars and the M_3_ occurs post‐weaning (Kierdorf & Kierdorf, 1989a), that is, when roe deer fawns living in a fluoride‐polluted environment feed entirely on contaminated plant material. The relationship between the developmental sequence of the permanent mandibular cheek teeth and their differential affection by fluorotic changes has led us to suggest that during the fetal and early postnatal periods of life, different mechanisms prevent the occurrence of markedly increased plasma fluoride levels in the fetus/fawn, thereby protecting its developing teeth against excessive fluoride exposure (Kierdorf, 1988; Kierdorf et al., 1991, 1993; Kierdorf & Kierdorf, 2000). The main protective mechanisms considered to be involved are a partial placental diffusion barrier to fluoride in the pregnant doe and a blood–milk barrier to fluoride in the lactating doe. These mechanisms normally prevent detrimental plasma fluoride concentrations in the fetus and in the fawn during the period of milk‐feeding (Figure 10). The rapid clearance of fluoride from plasma by the fast‐growing skeleton during the late fetal/early postnatal periods constitutes an additional factor contributing to the prevention of high plasma fluoride concentrations.

Variation in fluorosis severity among the mandibular cheek teeth similar to that described by us (Kierdorf, 1988; Kierdorf et al., 2012; Kierdorf & Kierdorf, 2000) was also observed in conspecifics living close to primary aluminum smelters in Norway (Vikøren & Stuve, 1996). In these roe deer, however, the M_1_ was sometimes severely fluorotic. Vikøren and Stuve (1996) attributed this to a fluoride disturbance of the late maturation stage of the M_1_ enamel in fawns living in a highly polluted habitat and ingesting very large amounts of fluoride with their initial plant diet.

Thus far, the existence of the postulated protective mechanisms has not been demonstrated experimentally in deer. However, experimental findings in cattle support the presence of a partial diffusion barrier to fluoride in the (epitheliochorial) ruminant placenta (Shupe et al., 1992), and a limited transfer of fluoride from blood to milk has been observed in humans and cattle (Backer‐Dirks et al., 1974; Ekstrand et al., 1984). In addition, the high calcium content of milk is known to reduce fluoride bioavailability (Shulman & Vallejo, 1990). The effect of the transition from milk to plant feeding was also demonstrated in goat kids, whose plasma fluoride concentration increased more than threefold between postnatal day 4 and postnatal days 30–50 (Skotnicka et al., 2018).

**FIGURE 11:**
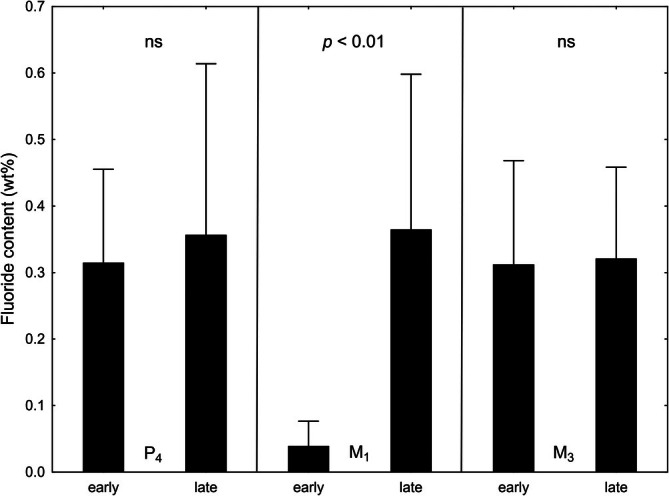
Comparison of the fluoride content of early and late formed dentin in P_4_, M_1_, and M_3_ of eight adult European roe deer with severe dental fluorosis (data from Richter et al., 2011). Values indicated are means and one standard deviation. The early formed dentin of the M_1_ shows a significantly (*t*‐test, *p* < 0.01) lower fluoride content than the late formed dentin of this tooth, while in the P_4_ and M_3_, the fluoride content of early and late formed dentin does not differ significantly (*t*‐tests, *p* < 0.05, ns) and is similar to that of the late formed dentin of the M_1_. This is evidence for a much lower plasma fluoride concentration during early (prenatal and early postnatal) dentin formation of the M_1_ compared to late dentin formation in this tooth and the entire period of dentin formation in the P_4_ and M_3_.

In the European roe deer, evidence in support of the postulated protective mechanisms comes from an electron‐microprobe study on the fluoride content of early‐formed (close to the EDJ) and late‐formed (juxtapulpal) dentin of P_4_, M_1_, and M_3_ of adult individuals with severe dental fluorosis (Richter et al., 2011). While in the M_1_, the fluoride content of the early‐formed dentin, that is, the dentin formed during late fetal and early postnatal life, was significantly lower (*t*‐test, *p* < 0.01) than that of the juxtapulpal dentin formed during later life, no significant differences (*t*‐tests, *p* > 0.05) were observed between these two dentin regions in the P_4_ and M_3_ (Figure 11). In the latter two teeth, crown formation takes place post‐weaning, that is, during a time when roe deer inhabiting fluoride‐polluted regions feed exclusively on (contaminated) plant material.

**FIGURE 12:**
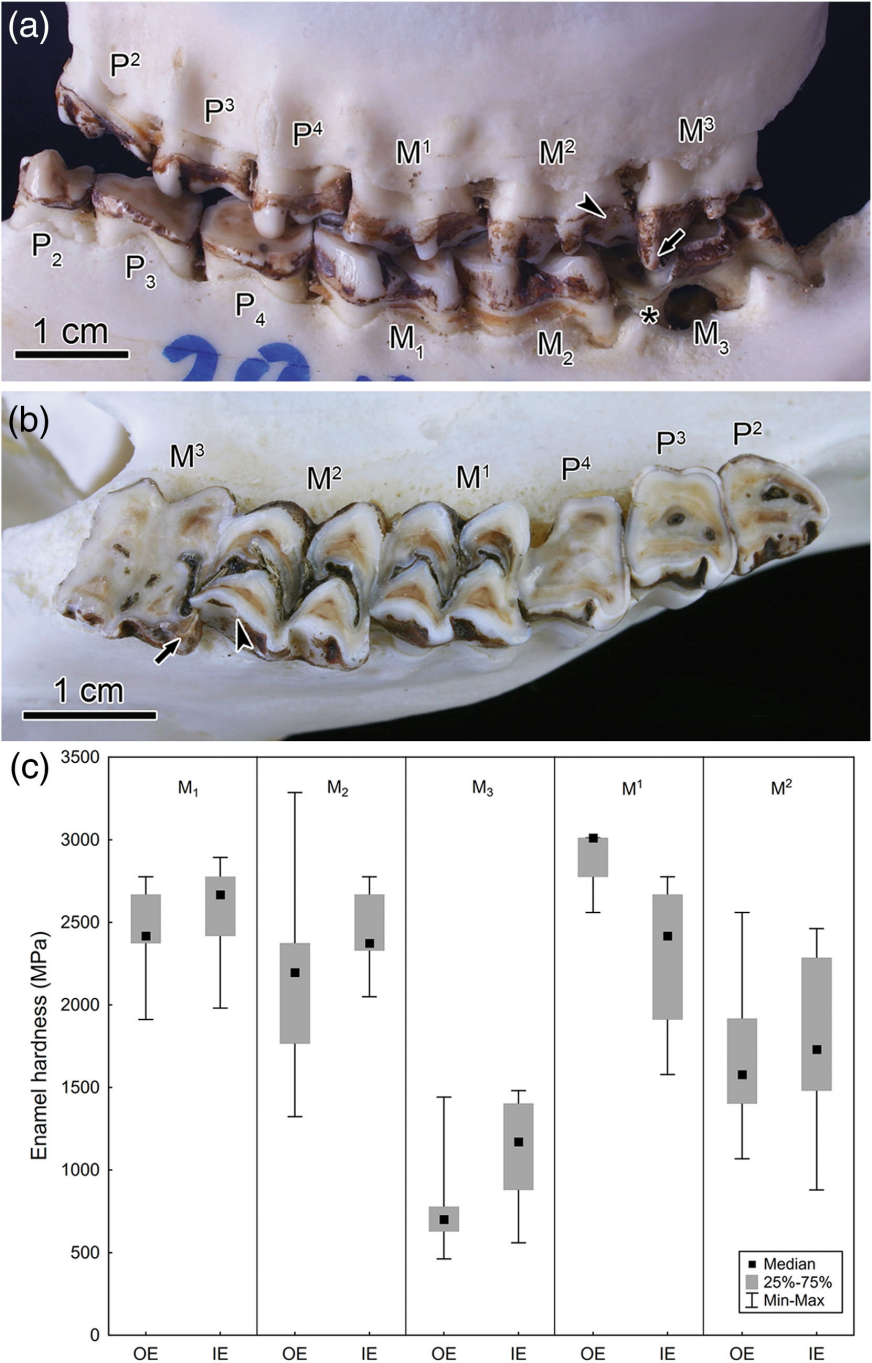
Macroscopic appearance of the fluorotic dentition of an approximately 8‐year‐old female roe deer from Slovenia and enamel hardness of its left maxillary and mandibular molars. (a) Buccal view of left maxillary and mandibular cheek tooth rows. Note pronounced reduction in height of the mesiobuccal cusp (protoconid) in the M_3_ (asterisk) and marked loss of alveolar bone in the region of the M_3_, as well as normal height of the distobuccal cusp (metacone, arrowhead) of the M^2^ and the mesiobuccal cusp (paracone, arrow) of the M^3^. (b) Occlusal view of left maxillary cheek tooth row, mesial to the right, buccal to the bottom of the image. Note intensified wear of P^2‐4^ and M^3^ as well as long M^2^ metacone (arrowhead) and M^3^ paracone (arrow). (c) Results of hardness testing of buccal (mandibular teeth) and palatal (maxillary teeth) enamel of the molars (Kierdorf & Kierdorf, unpublished data). Hardness testing was performed on polished sectional block surfaces (axiobuccolingual or axiobuccopalatal sections of resin‐embedded teeth). Prior to testing, the polished surface had been sputter‐coated with a thin layer (15 nm) of gold–palladium to increase the visibility of the indentations. These were positioned in outer enamel (OE, 50 μm deep to the outer enamel surface) and inner enamel (IE, 50 μm away from the EDJ) at 12 points located along the vertical axis of each tooth, using a Vickers diamond pyramid (load: 25 p, dwell time: 20 s). The M^3^ could not be analyzed because not enough palatal enamel had remained in this tooth. Note the much lower enamel hardness in the M_3_ compared to the M^2^, explaining the height reduction of the M_3_ protoconid.

Because hunters regularly collect roe deer mandibles for age estimation, mandibular tooth rows are more easily available than maxillary ones for assessing dental fluorosis. However, as is shown in Figure 12 for a fluorotic female individual with an estimated age of 8 years, also in maxillary dentitions, the permanent premolars and the third molar show more severe fluorotic changes, while the first molar is not or only slightly affected, and the second molar exhibits less intense fluorosis than P^2‐4^ and M^3^.

Figure 12a illustrates the pronounced reduction in height of the M_3_ protoconid, which has been recognized as a characteristic feature of this tooth in fluorotic roe deer dentitions. Figure 12a,b further demonstrates that the disto‐buccal cusp (metacone) of the M^2^ and the mesio‐buccal cusp (paracone) of the M^3^ are much less worn than the remaining crown portions of the respective teeth. This is regarded as a consequence of the more posterior location of the maxillary compared to the mandibular cheek teeth and the resulting occlusion of these two maxillary tooth cusps with the M_3_ protoconid. The intense wearing down of the latter cusp suggests that its enamel is less mineralized than that of (one of the) cusps of the occluding maxillary antagonists. The likely candidate is the metacone of the M^2^, since this tooth exhibits less severe fluorotic changes than the M^3^. This assumption was confirmed by hardness testing of the enamel of the three mandibular molars and the first and second maxillary molars of this individual (Figure 12c). The analysis further showed that especially the outer enamel of the M_3_ was severely hypomineralized. The characteristic pattern of dental wear observed in fluorotic roe deer dentitions thus reflects variation in enamel hardness of the occluding teeth. When the mesial portion of the M_3_ slides over the distal portion of the M^2^ during lateral jaw movement, the M_3_ protoconid becomes severely attrited by the more wear‐resistant metacone of the M^2^.

## 9 CONCLUSIONS AND OUTLOOK

**FIGURE 13:**
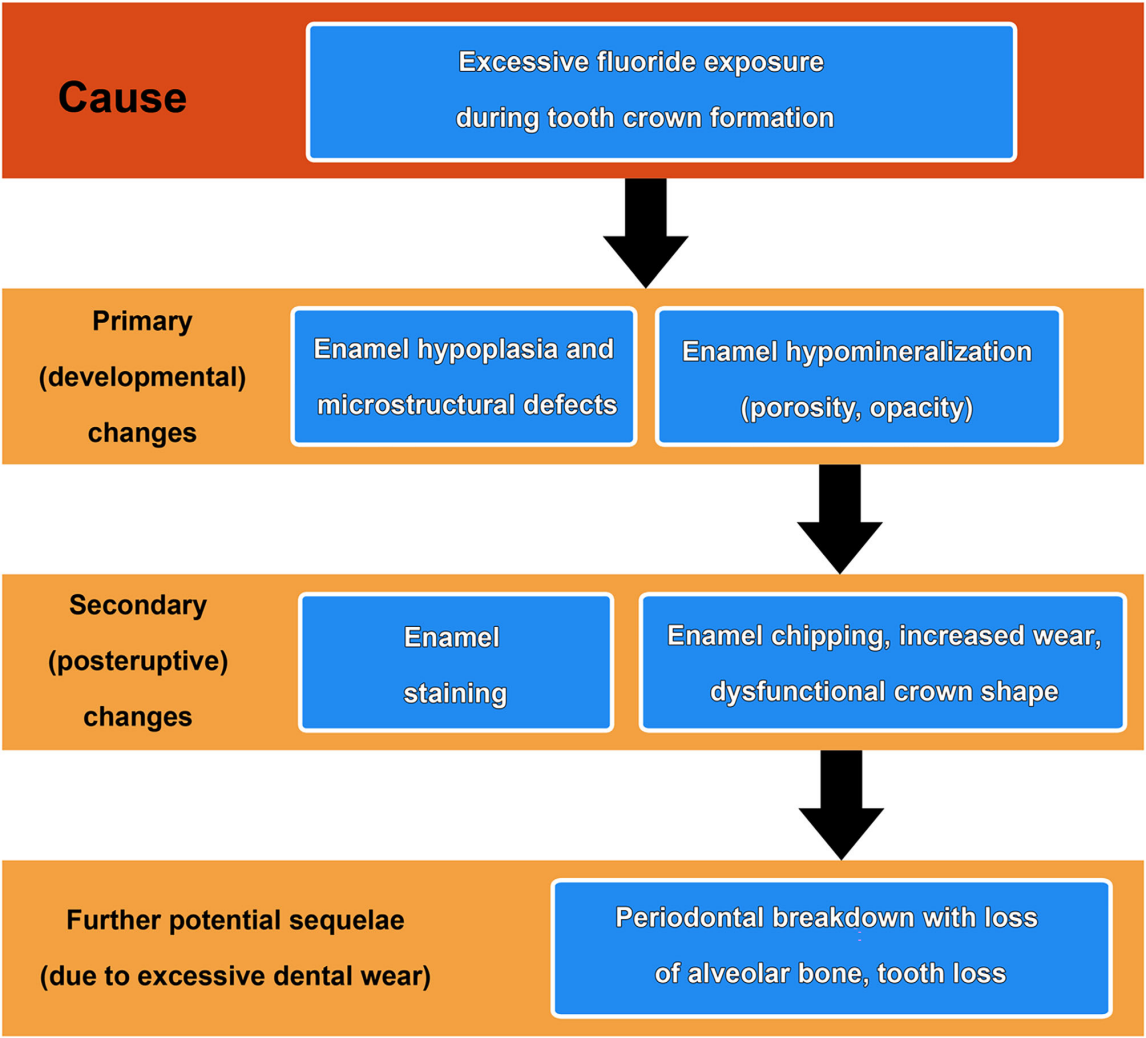
Schematic summary of the effects of excessive fluoride exposure during tooth formation on forming enamel and potential sequelae of these changes in affected teeth and dentitions.

The studies reviewed here provide a comprehensive survey of the macroscopic and histopathological features of dental fluorosis and the sequelae of these changes in a wild ruminant species (Figure 13). Moreover, a hypothesis explaining the variation of dental fluorosis severity among the permanent cheek teeth of the European roe deer has been put forward and tested.

Our results demonstrate that excess fluoride uptake impairs the secretory and maturation stages of amelogenesis. The primary (=developmental) changes seen in fluorotic enamel are structural defects (enamel hypoplasia, formation of prismless enamel) and enamel hypomineralization. Secondary changes developing as a consequence of this hypomineralization are posteruptive staining of the enamel and abnormally intense dental wear. Manifestations of excessive dental wear include a reduction in height or complete loss of the enamel ridges on the occlusal surface of the affected teeth, the occurrence of posteruptive enamel defects (enamel chipping), premature reduction of crown height, and the formation of abnormally shaped tooth crowns. A further potential sequela, resulting from the abnormal tooth wear, is periodontal breakdown with alveolar bone loss, which in severe cases can lead to tooth loss. There is evidence from North American wapiti that the impairment of dental function due to dental fluorosis causes early senescence and a reduced life expectancy (Garrott et al., 2002). For the European roe deer, a species that is managed by hunting all across its range (Lorenzini et al., 2022), such data are currently not available.

The variation in fluorosis severity among the permanent cheek teeth of the European roe deer has been related to the developmental sequence of its dentition. It has been hypothesized that protective mechanisms (placental barrier, blood–milk barrier) prevent excess plasma fluoride levels during the prenatal and early postnatal periods in animals from fluoride‐polluted environments. Support for this hypothesis comes from studies using the fluoride content of dentin formed during different ontogenetic periods (Richter et al., 2011).

Our hypothesis explaining the distribution of dental fluorosis in the permanent cheek teeth of the European roe deer has been extended to red deer by Launer et al. (1996). However, we have shown that the red deer M_2_ is more intensely affected by fluorosis compared to that of the roe deer (Kierdorf & Kierdorf, 2000). This can be attributed to the fact that crown formation of the M_2_ in the red deer occurs completely post‐weaning, while in the roe deer, only the later stages of M_2_ crown formation take place after weaning (Kierdorf & Kierdorf, 2000). This highlights the necessity to address the distribution of dental fluorosis in the dentition of wild ruminants on a species‐by‐species basis, taking into account interspecific variation in the timing of dental development in relation to birth and weaning. Further studies on other deer species from regions with high fluoride levels are therefore encouraged.
